# Supplementary material for: From sequence to enzyme mechanism using multi-label machine learning
Source: BMC Bioinformatics. 2014 May 19;15:150. doi: 10.1186/1471-2105-15-150 (PMC4229970; doi:10.1186/1471-2105-15-150)
Supplement: Additional file 2 — Java code of ml2db. Additional file ml2db_code.tar.gz contains the Java source code to run the multi-label machine learning experiments and save the results to database. The code’s Javadoc is included. [file 1471-2105-15-150-S2.zip › additional file 2/ml2db/utils/doc/uk/ac/standrews/utils/main/database/DbWriter.html]

DbWriter


JavaScript is disabled on your browser.


- Overview
- Package
- Class
- Use
- Tree
- Deprecated
- Index
- Help

- Prev Class
- Next Class

- Frames
- No Frames

- All Classes

- Summary:
- Nested |
- Field |
- Constr |
- Method

- Detail:
- Field |
- Constr |
- Method


uk.ac.standrews.utils.main.database

## Class DbWriter

- java.lang.Object
- - uk.ac.standrews.utils.main.database.DbManaged
  - - uk.ac.standrews.utils.main.database.DbWriter

- ---

    

  ```
  public class DbWriter
  extends DbManaged
  ```

  Provides methods to write to a database.
  Provides generic query and table create/drop methods.

  Version:
  :   20 Feb 2009

  Author:
  :   Luna De Ferrari luna.deferrari-at-ed.ac.uk

- - ### Constructor Summary

    Constructors

    Constructor and Description

    `DbWriter(DbManager dbManager)`
  - ### Method Summary

    Methods

    | Modifier and Type | Method and Description |
    | --- | --- |
    | `boolean` | `executeUpdate(java.lang.String sqlToExecute)` Executes any SQL update which does not return results (create/drop table etc) |

    - ### Methods inherited from class uk.ac.standrews.utils.main.database.DbManaged

      `getDbConnection, getDbManager, getDbReader`
    - ### Methods inherited from class java.lang.Object

      `equals, getClass, hashCode, notify, notifyAll, toString, wait, wait, wait`

- - ### Constructor Detail


    - #### DbWriter

      ```
      public DbWriter(DbManager dbManager)
      ```
  - ### Method Detail


    - #### executeUpdate

      ```
      public boolean executeUpdate(java.lang.String sqlToExecute)
      ```

      Executes any SQL update which does not return results (create/drop table
      etc)

      Parameters:
      :   `sqlToExecute` - sql code to execute

      Returns:
      :   true if the sql command was executed


- Overview
- Package
- Class
- Use
- Tree
- Deprecated
- Index
- Help

- Prev Class
- Next Class

- Frames
- No Frames

- All Classes

- Summary:
- Nested |
- Field |
- Constr |
- Method

- Detail:
- Field |
- Constr |
- Method
